# Mind the Gap: A Systematic Review of Borderline Intellectual Functioning in Childhood and Adolescence

**DOI:** 10.1155/bn/9979097

**Published:** 2026-04-29

**Authors:** Paolo Stievano, Valeria Mammarella, Carlo Di Brina

**Affiliations:** ^1^ Department of Mental Health; Department of Psychology, Sapienza University of Rome, Rome, Italy, uniroma1.it; ^2^ Unità Operativa Complessa Neuropsichiatria Infanzia e, Adolescenza Azienda Sanitaria Locale Roma 2, Rome, Italy; ^3^ Department of Psychology, Sapienza University of Rome, Rome, Italy, uniroma1.it; ^4^ Unit of Child Neurology and Psychiatry, Department of Human Neuroscience, Sapienza University of Rome, Rome, Italy, uniroma1.it

## Abstract

**Background:**

Borderline intellectual functioning (BIF) is characterized by an IQ between 70 and 84/85, representing a cognitive condition associated with significant learning, academic, and behavioral challenges. Despite its prevalence and impact, BIF remains underrecognized in diagnostic classifications, leading to inconsistent clinical management and limited research attention.

**Objectives:**

This systematic review is aimed at synthesizing the current literature on BIF in childhood and adolescence, focusing on its neuropsychological profiles, prevalence, and effective strategies to improve quality of life.

**Methods:**

We conducted a systematic review following the PRISMA 2020 guidelines. Articles published between 2013 and 2023 were identified through electronic databases, including MEDLINE, PsycInfo, and CINAHL. Inclusion criteria were studies presenting empirical data on BIF in individuals aged 0–18 years, with quantitative results on intellectual and adaptive functioning. Excluded were intervention studies, reviews, and studies focusing on unrelated neuropsychological domains.

**Results:**

From 455 articles identified, eight met the inclusion criteria. Key findings include that BIF is associated with cognitive deficits, particularly in working memory and academic skills, with a 2‐year developmental lag in reading and arithmetic. Prevalence estimates range from 7% to 14%, influenced by socioeconomic factors and environmental stressors. Tailored educational programs and vocational training are effective in addressing cognitive and adaptive challenges, highlighting the need for personalized interventions.

**Conclusions:**

BIF presents significant clinical, educational, and social challenges that require standardized diagnostic criteria and targeted interventions. Future research should focus on refining diagnostic approaches, exploring cognitive subtyping, and implementing cost‐effective support systems to enhance the well‐being of individuals with BIF.

## 1. Introduction

### 1.1. Definitions and Diagnosis Criteria

Borderline intellectual functioning (BIF) is a cognitive condition defined by an intelligence quotient (IQ) range of 70–85, situating individuals in a transitional zone between average cognitive functioning and intellectual disability. This IQ range, which falls one to two standard deviations below the average, leads to learning, academic, and behavioral challenges, including difficulties with attention. Although BIF is not classified as an intellectual disability, it significantly impacts children′s lives, affecting academic performance, social integration, and overall quality of life. Despite its considerable implications, BIF has received limited attention in scientific literature and clinical practice, contributing to gaps in diagnosis and intervention [1, 2].

The complexities of diagnosing BIF are exacerbated by the lack of standardized diagnostic criteria across classification systems such as the DSM‐5 and ICD‐11. The DSM‐5, published in 2013 by the American Psychiatric Association, mentions BIF only in a supplementary V‐code section, whereas neither ICD‐10 nor ICD‐11 provides a designated category for this condition. This inconsistency in diagnostic guidelines creates challenges for clinicians in accurately diagnosing BIF and determining when intervention is necessary [2]. Recent changes in diagnostic frameworks, particularly the extension of the developmental period to 22 years, underscore the importance of continued support as individuals transition into adulthood [3]. This change recognizes that developmental challenges may not end at 18, as traditionally believed, but can extend into the early 20s. Acknowledging this longer developmental period highlights the need for a more holistic approach to intervention and support, ensuring that individuals with BIF receive the necessary assistance throughout their formative years and as they move into adulthood. In this context, recent work has further emphasized that BIF should not only be conceptualized exclusively through IQ thresholds, but also in relation to adaptive functioning across conceptual, social, and practical domains [4].

Furthermore, the absence of a universally accepted term for BIF complicates communication among healthcare professionals and educators, potentially leading to the stigmatization of affected individuals [1]. Individuals with BIF often experience cognitive deficits, academic challenges, and difficulties in adaptive behavior, which can negatively impact their social, academic, and occupational performance. The prevalence of BIF varies among studies and is influenced by factors such as socioeconomic status and environmental stressors [1, 2]. BIF is part of the typical IQ distribution and may be influenced by genetic factors or specific disorders, including 22q11 deletion syndrome, fragile X premutation, neurofibromatosis Type 1, and fetal alcohol spectrum disorders [5].

Given these complexities, it is essential to carefully assess multiple variables to develop personalized intervention strategies. Co‐occurring mental health conditions further necessitate comprehensive and tailored approaches [6]. Additionally, parents frequently encounter emotional challenges, such as anxiety, uncertainty, and defensive responses, when navigating diagnostic and intervention procedures. Providing comprehensive support systems and clear information about BIF, potential interventions, and available resources is vital [7].

### 1.2. Prognostic and Therapeutic Approaches

Research indicates that children with BIF exhibit cognitive developmental lags of less than 1 year compared with their age‐matched peers, accompanied by a 2‐year academic lag in arithmetic and reading skills [8] Despite these delays, their developmental growth trajectories are similar to those of their age‐matched counterparts [8] . When assessing academic abilities, it is crucial to offer tasks that are appropriate for the child′s grade level and age while also seeking a level compatible with the child′s abilities to accurately assess the state of their learning. This approach allows for the identification of the child′s proximal zone of development, which is essential for potential interventions aimed at enhancing their academic skills. It supports the gradual and complete achievement of educational goals by reducing stress and appropriately challenging the child′s capabilities.

Importantly, this does not mean an automatic lowering academic goal; instead, it suggests implementing differentiated interventions tailored to each child′s individual needs [8] . This highlights the importance of designing personalized educational strategies to address specific weaknesses in mathematics and reading skills. Additionally, it is important to recognize that the diagnostic category of BIF is complex, necessitating further research and reflection to better understand and define the condition [9].

## 2. Rationale of the Review

Our study examined the features and shared characteristics of individuals diagnosed with BIF with the goal of enhancing understanding and informing clinical management, particularly during early development. By conducting a thorough analysis of the relevant literature on various aspects of BIF in childhood and adolescence, we aim to provide insights into the clinical implications, diagnostic challenges, and impacts on affected individuals and their families. This review is aimed at bridging these gaps by offering a comprehensive analysis of BIF, focusing on diagnostic challenges, cognitive and developmental profiles, prognostic implications, and educational strategies. Clear definitions are crucial in ensuring that individuals with BIF receive the support and respect they deserve, promoting inclusivity and fostering supportive environments.

To understand the multifaceted nature of BIF and its implications across clinical, educational, and epidemiological domains, we focused on three main areas of interest. First, we examined the intellectual and neuropsychological profiles of children with BIF. In this review, the term neuropsychological profile refers to the organization of cognitive functioning across specific domains. Beyond the five domains identified by Lezak et al. [10], global IQ, executive functions, memory, language, and sociocognitive skills, in developmental clinical practice, it is customary to include the praxic domain and academic learning as well [11]. This framework allows a structured comparison of findings across studies, moving beyond global intelligence scores and providing a more nuanced characterization of strengths and weaknesses in BIF. Second, we explored the prevalence and epidemiology of BIF, including population size and distribution. Third, we reviewed strategies aimed at improving individual well‐being, such as educational and vocational programs designed to enhance quality of life.

### 2.1. Methods

To conduct this systematic review, we followed the recommended reporting criteria for systematic reviews and meta‐analyses (PRISMA 2020) [12, 13].

### 2.2. Eligibility Criteria

To select suitable publications for the present review, we established the following inclusion criteria: (a) publication in English, (b) papers presenting original and empirical data on BIF in developmental age (0–18), (c) studies conducted both in clinical and community settings, and (d) studies reporting quantitative results on multiple domains of intellectual functioning in this population. We excluded intervention studies and studies in which only other neuropsychological domains were assessed (e.g., attention, memory, and language), studies on intellectual disability and BIF data, mixed and indiscernible studies, and studies on populations with psychiatric or neurological disorders. We did not consider the following article types: case reports/series, lectures, book chapters, editorials, commentaries, and review articles.

### 2.3. Information Sources

Studies published between January 2013 and September 2023 were searched using the following electronic databases: EBSCO, MEDLINE, APA PsycInfo, CINAHL Plus, Psychology and Behavioral Sciences Collection, ERIC, and APA PsycArticles. Other sources of eligible articles were the reference lists of the review articles. The last search date was on September 7, 2023. The search was updated to include studies published in 2024 and 2025; the updated search was run on January 14, 2026, and covered the period from September 8, 2023, to December 31, 2025.

### 2.4. Search Strategy

The following keywords were used to perform the search in all databases: “borderline intellectual functioning,” “borderline intelligence,” “intellig∗”, “intellect∗”. The exact search string used for all databases consulted was: AB/(“borderline intellectual functioning” OR “borderline intelligence” OR “intellect∗” OR “intellig∗”) AND AB/(“child∗” OR “adolesc∗” OR “development∗” OR “young” OR “infant∗”), so that the search of the string was limited to abstracts. We also limited the search to studies published between January 2013 and the last date of the search, excluding commentaries, case reports/series, letters to the editor, book chapters, editorials, and communication articles. We did not include review articles as eligible studies; nevertheless, we screened them to retrieve additional original empirical studies from their reference lists.

### 2.5. Selection Process

Two reviewers independently screened each record by title and abstract, including only records relevant to BIF in the infant and adolescent populations. Subsequently, the same two reviewers independently excluded papers according to the eligibility criteria by screening the full texts of the articles, and for each, they separately documented the reasons for exclusion. At the end of this phase, they discussed disagreements, and if a consensus could not be reached, they referred to a third reviewer to make the final decision.

### 2.6. Data Collection Process and Items

We extracted data from the articles using Excel spreadsheets. Each of the two reviewers screened half of the articles and rechecked the papers assigned to the others. A third reviewer double‐checked all extracted data. Data items were established according to the PICO framework, including general article information (title, authors, journal, publication year, and study type), study design, country, participant ethnicity, socioeconomic status, setting, population features (experimental sample size, age, gender, and control group characteristics if present), inclusion criteria, diagnosis and diagnostic methods, outcome measures (IQ indexes, executive functions, and adaptive level), results, potential biases, funding sources, and conflicts of interest.

### 2.7. Synthesis Methods

Qualitative data synthesis was conducted using textual descriptions and thematic analysis. The findings are summarized in Tables 1 and 2 (studies) and 3 (levels of evidence). To support interpretation of the findings, we classified the methodological strength of each included study according to the Oxford Centre for Evidence‐Based Medicine (OCEBM) Levels of Evidence framework. Levels were assigned based on study design (e.g., longitudinal vs. cross‐sectional), the presence of a comparison/control group, and key methodological features. These levels should be interpreted as an indicator of the robustness of the available evidence and not as a quantitative measure of effect.

**Table 1:** Characteristics of the included studies.

Title	Publication year	Study design	Author	Location	Diagnosis method	Inclusion criteria	*N* experimental	Average age experimental group (M in months and years)	Sex experimental group
Development of Cognitive Functions and Academic Skills in 9‐ to 10‐Year‐Old Children With Borderline Intellectual Functioning	2021	LS	Träff, Ulf; Östergren, Rickard	Community setting	PM Raven, linguistic test	Swedish native language, normal/corrected visual acuity, no hearing impairment.	27 subjects	126, SD 7.23 months	37% M, 63% F
Intellectual Profile in School‐Aged Children With Borderline Intellectual Functioning	2019	OS	Pulina, Francesca; Lanfranchi, Silvia; Henry, Lucy; Vianello, Renzo	Community setting	WISC IV	QI, grades (primary and lower secondary school)	204 subjects	119, SD 28 months	68% M, 32% F
Intervening on the Developmental Course of Children With Borderline Intellectual Functioning With a Multimodal Intervention: Results From a Randomized Controlled Trial	2020	RCT	Blasi, Valeria; Zanette, Michela; Baglio, Gisella; Giangiacomo, Alice; Di Tella, Sonia; Canevini, Maria Paola; Walder, Mauro; Clerici, Mario; Baglio, Francesca	Outpatient	WISC III	FS IQ score: 70 85 (±5) with assessed learning disabilities	36 subjects	96, SD 1.46 months	22% M, 78% F
Meeting the Support Needs of Persons With Mild Intellectual Disability or Borderline Intellectual Functioning: Still a Long Way to Go	2017	RS	Ouwens, P. J. G.; Smulders, N. B. M.; Embregts, P. J. C. M.; van Nieuwenhuizen, C	Outpatient	Not specified	Individuals with MID (IQ:50–69)or BIF (IQ:70–85)	250 subjects	26.1 SD 13.8 range 3–65 years	61% M, 39F%
Memory and Linguistic/Executive Functions of Children With Borderline Intellectual Functioning	2019	CS	Água Dias, Andrea B.; Albuquerque, Cristina P.; Simões, Mário R.	Community setting	WISC III	Full scale IQs between 70 and 85	40 subjects	123.6 SD 26,136 months	40% M, 60% F
Social Competence in Children With Borderline Intellectual Functioning: Delayed Development of Theory of Mind Across All Complexity Levels	2016	OS	Baglio, Gisella; Blasi, Valeria; Intra, Francesca Sangiuliano; Castelli, Ilaria; Massaro, Davide; Baglio, Francesca; Valle, Annalisa; Zanette, Michela; Marchetti, Antonella	Community setting	WISC III	BIF with IQ scores ranging from 70 to 85 were included	28 subjects	113.52 SD 15.12	57% M, 43% F
Stability of WISC‐R Scores in Students With Borderline Intellectual Functioning	2014	LS	Jankowska, Anna Maria; Bogdanowicz, Marta; Takagi, Anna	Outpatient	WIsc‐R	−1.01 and −2.00 (standard deviations)	30 subjects	Three times: at approximately 8, 10.8, and 13.6 years old	77%M, 23% F
Challenges and Neuropsychological Functioning in Children and Adolescents With Borderline Intellectual Functioning	2022	RCR	Heli Sätilä, Laura Mirjami Jolma, Mira Meriläinen‐Nipuli, and Mikko Koivu Jolma.	Outpatient	Nepsy‐II, Wpps‐III	BIF: Delay ≥ 1.5 SD below mean in 2+ developmental domains.	651 subjects	Mean: 164.4 (range: 5.2–27), 68% under 192 months, 32% over 192 months; SD not provided.	

Abbreviations: CS, cross‐sectional study; LS, longitudinal study; OS, observational study; RCR, retrospective chart review; RCT, randomized controlled trial; RS, retrospective study.

**Table 2:** Intelligence quotients (IQ) variations among the studies included.

Title	QI indexes results experimental group	*N* controls (if present)	QI indexes results control group	Outcome measures	Results
Development of Cognitive Functions and Academic Skills in 9‐ to 10‐Year‐Old Children With Borderline Intellectual Functioning	Raven percentiles 10.63 M, 3.34 DS; verbal ability 10.34, 3.33 DS	28 subjects	Raven 49,77 percentiles, 9.64 DS; verbal ability 50.05, 14.24	Processing speed (digit matching), executive functioning, shifting (trial making), semantic fluency, phonological fluency, visual spatial wm, maths, and reading tests	
Intellectual Profile in School Aged Children With Borderline Intellectual Functioning	Mean full scale IQ between 70 and 85, no standardized assessment of adaptive functioning	60 subjects	Mean full scale IQ 100.63 DS 11.63	Wisc IV indexes and FSQI (vci, pri, wmi, and psi), Gai, and cpi. With comorbidities and without	
Intervening on the Developmental Course of Children With Borderline Intellectual Functioning With a Multimodal Intervention: Results From a Randomized Controlled Trial	IQ, M‐ABC; Vineland II, CBCL, and neuropsychological data	18 subjects	IQ, M‐ABC; Vineland II, CBCL, and neuropsychological data	WISC III, Child Behavior Checklist (CBCL), Vineland II, and Movement ABC	
Meeting the Support Needs of Persons With Mild Intellectual Disability or Borderline Intellectual Functioning: Still a Long Way to Go	The majority (56.5%) of the participants had BIF, and the remainder (43.6%) had MID (*χ*2(1) = 245.95, *p* = 0.00).	—	—	A normative and objective assessment of support needs	
Memory and Linguistic/Executive Functions of Children With Borderline Intellectual Functioning	The study does not provide information on the specific IQ indexes results for the experimental group	—		The study assessed verbal memory, visual memory, memory‐related tasks, executive functions, and language skills in children with borderline intellectual functioning (BIF) compared with a control group. Outcome measures included tests such as Word List, Narrative Memory, Face Memory, Rey Complex Figure, and Verbal Fluency. These measures evaluated domains like language, memory, and attention/executive functions.	Children with BIF displayed deficits in short‐term verbal memory and interference effects, along with impaired long‐term verbal memory in older children. Difficulties were noted in recalling and retelling oral stories, whereas long‐term visual memory remained intact. They also exhibited coordination difficulties in cognitive processes, impacting rapid naming and phonemic fluency, which could affect written language. Correlations between WISC‐III scores and neuropsychological test scores were generally low, emphasizing the need for both types of measures.
Social Competence in Children With Borderline Intellectual Functioning: Delayed Development of Theory of Mind Across All Complexity Levels	FSIQ 78.61 (3.87)	31 subjects	FSIQ 105.36 (10.38)	Memory assessment: Word List, Visual Recognition Span, Corsi Block‐Tapping. Attention and executive functions assessment: Trail Making Test, and Digit Span, Verbal Fluency.	
Stability of WISC‐R Scores in Students With Borderline Intellectual Functioning	QIT:1) 77.83 SD 7.86; 2) 79.48 SD 8.67; 3) 78.10 SD 4.82			Global IQ comparison over time; verbal IQ versus nonverbal IQ comparison; verbalsubitem versus nonverbal subitem change comparison.	Stability observed in Full scale over time. Decrease in VIQ, increase in PIQ.
Challenges and Neuropsychological Functioning in Children and Adolescents with Borderline Intellectual Functioning	Not explicitly stated			Changes in neuropsychological functioning, as well as conduct, self‐esteem, and activities of daily living.	

**Table 3:** Levels of evidence of included studies.

Title	Level of evidence	Evidence level analysis
Development of Cognitive Functions and Academic Skills in 9‐ to 10‐Year‐Old Children With Borderline Intellectual Functioning	Level 2	Analytical observational study with statistical analysis.
Intellectual Profile in School‐Aged Children With Borderline Intellectual Functioning	Level 3	Observational study with control group and standardized assessments.
Intervening on the Developmental Course of Children With Borderline Intellectual Functioning With a Multimodal Intervention: Results From a Randomized Controlled Trial	Level 1	Randomized controlled trial evaluating intervention effectiveness.
Meeting the Support Needs of Persons With Mild Intellectual Disability or Borderline Intellectual Functioning: Still a Long Way to Go	Level 3	Epidemiological study on prevalence and support needs.
Memory and Linguistic/Executive Functions of Children With Borderline Intellectual Functioning	Level 3	Observational study assessing cognitive and executive functions.
Social Competence in Children With Borderline Intellectual Functioning: Delayed Development of Theory of Mind Across all Complexity Levels	Level 3	Observational study comparing theory of mind development.
Stability of WISC‐R Scores in Students With Borderline Intellectual Functioning	Level 3	Longitudinal cohort study on WISC‐R score stability.
Challenges and Neuropsychological Functioning in Children and Adolescents With Borderline Intellectual Functioning	Level 4	Retrospective clinical study of neuropsychological functioning.

## 3. Results

### 3.1. Study Selection

**Figure 1:**
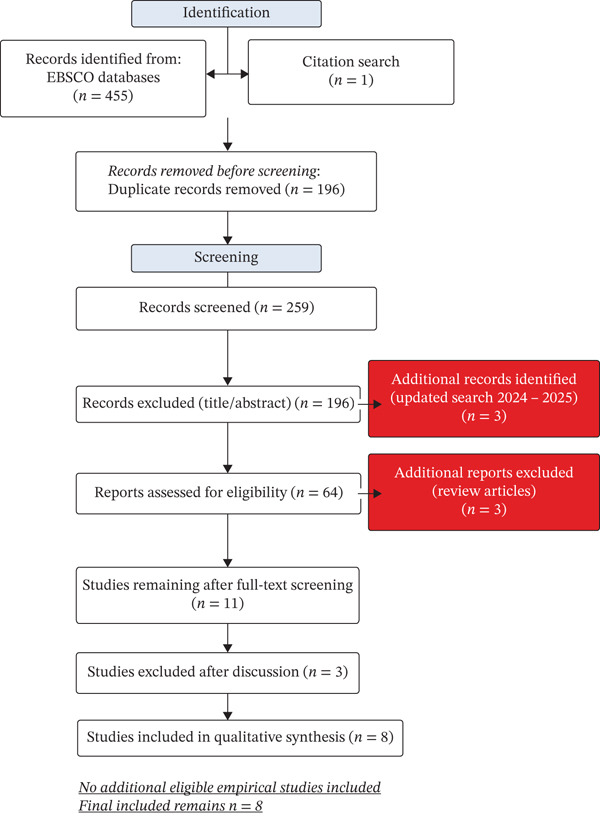
PRISMA 2020 flow diagram illustrating the study selection process for the systematic review on borderline intellectual functioning in children and adolescents. The diagram shows the original database search (January 2013–September 2023) and an updated search (September 2023–December 2025), with 456 records identified (455 from databases and one from citation search), 196 duplicates removed, 259 records screened (196 excluded at title/abstract stage), 64 full‐text articles assessed (53 excluded) and 11 articles initially retained (three later excluded after discussion), resulting in eight studies included in the review. The updated search identified three additional records (all excluded as reviews/synthesis), leaving a final total of eight included studies.

Our systematic search of the EBSCO databases identified 455 articles, of which 196 were deleted as duplicates. From the remaining 259 articles, we excluded 196 by title and abstract because they did not meet the inclusion criteria or were out of theme. Another one record was identified through citation search. Consequently, 64 articles were assessed for eligibility, of which 53 were excluded for reasons reported in the PRISMA 2020 flow diagram (Figure 1). From the remaining 11 studies, we excluded three that did not meet all the inclusion criteria after a discussion between the two reviewers responsible for the selection process. Thus, the remaining eight articles were included in this review. The updated search (2024–2025) identified three additional records. After full‐text screening, these papers were excluded because they were review/synthesis articles and therefore did not meet the eligibility criterion requiring original empirical data. These recent contributions were retained as background references to update the conceptual discussion.

### 3.2. Study Characteristics

The reviewed studies encompassed a diverse range of methodologies and focused on the areas related to BIF. Various diagnostic criteria and assessment tools have been utilized across studies, with most employing the Wechsler Intelligence Scale for Children (WISC) (WISC‐R, WISC‐III, and WISC‐IV) to assess general intelligence and to identify cognitive profiles. However, some studies used neuropsychological tests to explore specific cognitive domains. Notably, longitudinal studies, such as those by Träff and Östergren, have provided valuable insights into the developmental trajectory of children with BIF, highlighting cognitive weaknesses and academic challenges compared with typically developing peers.

### 3.3. Results of Synthesis

Analysis of the results from the reviewed studies demonstrates the complexity of BIF and the challenges in identifying it, estimating its prevalence, and developing effective interventions. Despite varying definitions and diagnostic criteria, researchers have agreed that individuals with BIF experience cognitive deficits and academic difficulties. To improve quality of life and social integration, targeted interventions such as personalized educational programs, vocational training, and psychological support should be implemented.

#### 3.3.1. Intellectual and Neuropsychological Profiles of Children

Multidisciplinary assessments conducted by specialists were used to evaluate and characterize the intellectual and neuropsychological profiles of the children with BIF. In a study conducted by Sätilä et al. [14], BIF was diagnosed through comprehensive evaluations performed by a team of specialists including pediatric neurologists, neuropsychologists, speech therapists, occupational therapists, and physiotherapists. Children who received the ICD‐10 diagnosis of F83 (mixed specific developmental disorder), displayed a delay of 1.5 or more standard deviations below the predicted scores by age, in two or more developmental domains, including gross and fine sensorimotor skills, language, cognition, socioemotional development, or daily activities [14].

A neuropsychologist and a pediatric neurologist assessed school‐aged individuals with learning difficulties. According to the ICD‐10 diagnosis of F81.3 (mixed disorder of scholastic skills), when two or more cognitive domains are delayed by at least one to two standard deviations below the mean in standardized tests but do not meet the criteria for intellectual disability, and the child′s situation is not caused by a poor learning environment or psychiatric disorder, the diagnosis is assigned [14]. The standardized tests used in the assessment included the Neuropsychological Test for Children (NEPSY‐II), the Wechsler Preschool and Primary Scale of Intelligence (WPPSI‐III)/Wechsler Intelligence Scale for Children (WISC‐IV), and, if appropriate, the Attention‐Deficit Hyperactivity Disorder (ADHD) Rating Scale‐IV, the Finnish version of the Social Responsiveness Scale (SRS), and the Strengths and Difficulties Questionnaire (SDQ‐Fin) [14]. Comorbidities such as ADHD or autism spectrum disorder were diagnosed according to the ICD‐10 classification of Sätilä [14].

The definition and diagnostic criteria for BIF may vary slightly across studies but generally include an IQ in the lower range of normalcy (70–85) and the presence of difficulties in achieving academic goals and adaptive behavior. Most studies have used tools such as the WISC to assess general intelligence and identify specific cognitive profiles. Träff and Östergren conducted a longitudinal study of 310 children from 30 schools, all of whom had Swedish as their native language, normal vision, and no hearing loss. The assessment utilized Raven′s Standard Progressive Matrices and Järpsten and Taube′s word ability test to determine nonverbal and verbal IQ percentiles [8].

A study conducted by Träff and Östergren [8] showed that children with BIF, except for the 2 years delay, have cognitive development patterns similar to those of their peers without neurodevelopmental disorders. However, Vianello et al. [9] reported a diverse cognitive profile on the WISC‐IV, with lower scores on working memory and higher scores on perceptual reasoning. This profile is distinct not only from that of typically developing children but also from those with other neurodevelopmental disorders. Despite comorbid neurodevelopmental disorders, the cognitive profile of children with BIF remains unchanged. Träff and Östergren [8] found that these children have weaknesses in academic skills, such as arithmetic and reading tasks, and a slower rate of word‐reading development than their peers. This highlights the importance of longitudinal research to understand the cognitive and academic development of children with BIF.

The research conducted by Dias et al. [15] identified the cognitive profiles associated with BIF in school‐aged children. The study showed deficits in neuropsychological areas when comparing children with BIF with a control group. These deficits include a lack of short‐term verbal memory and an interference effect, impairment in long‐term verbal memory, particularly in older children, and difficulties in recalling and retelling oral stories. Additionally, coordination difficulties across various cognitive processes were observed, as evidenced by performance on the rapid naming tests and deficits in phonemic fluency. These deficits, in conjunction with poor performance in rapid naming, may negatively affect writing skills.

Literature indicates that both the WISC‐III and neuropsychological tests are important because of their low correlations. However, the stability of the WISC‐R results over time in students with BIF has been inconsistent. Whereas some studies suggest that low stability leads to fluctuations in the IQ [16], others show high stability despite variations in fluid intelligence scores [15, 16]. This highlights the cognitive challenges faced by children with BIF, especially with regard to memory, language, and executive function. Children with BIF also experience a delay in the development of the theory of mind (ToM) compared with typically developing children [17]. Specifically, they perform poorly in first‐ and second‐order ToM tasks and advanced ToM tasks compared with their typically developing counterparts. Executive function and general intelligence may also influence ToM performance in children with BIF. Therefore, it is crucial to consider ToM development in assessments and early interventions to enhance social competence in these children.

#### 3.3.2. Prevalence and Epidemiology

Investigating the number of people affected by BIF, recent studies estimated that the prevalence of BIF ranges from 7% to 14% [18], [9]. This condition may be influenced by various social and environmental factors such as low socioeconomic status, maltreatment, and high levels of maternal stress. Baglio et al. [17] state that the prevalence of BIF varies and there is no international consensus on diagnostic criteria, making it difficult to precisely determine the prevalence of this condition. However, some studies have reported a frequency of BIF ranging from 2% to 7%–18% in the general population and approximately 7% among school‐aged children.

Nouwens [19] asserted that the prevalence and epidemiology of BIF are topics of interest for understanding how many individuals are affected by this condition and how it is distributed in the population. Dias [15] emphasized that owing to the lack of uniformity in the terminology and classification of intellectual disorders, accurately determining the prevalence of BIF can be complex without a clear definition. Jankowska [16] highlighted that some studies based on a normal distribution curve estimated that approximately 14% of the population might have BIF. However, the lack of consistency in the diagnostic criteria and variations in the assessment methods can influence the estimation of the actual prevalence of BIF.

Sätilä [14] highlighted that the prevalence and epidemiology of BIF are topics of interest to understand how many individuals are affected by this condition and how it is distributed in the population. Key points to consider include prevalence, population distribution, geographical variation, and implications for public health.

#### 3.3.3. Educational Measures and Clinical Interventions Aimed at Enhance the Quality of Life of Individuals With BIF

Träff and Östergren [8] highlighted the need for tailored interventions to improve the learning and development of these children. Without proper support, they risk dropping out of school, which can have long‐term negative effects on their educational prospects. To help children with BIF catch up, educators may need to provide additional learning time and resources, such as extended learning opportunities, remedial classes, or individual tutoring, and address behavioral, social, and emotional difficulties. Implementing personalized educational and vocational training programs can improve cognitive and social skills, facilitate employment, and promote overall well‐being, enabling individuals with BIF to realize their full potential and participate actively in society [9].

Recognizing the high prevalence of comorbid neuropsychiatric and psychiatric diagnoses among individuals with BIF can help develop personalized support plans that cater to their specific needs, as per this study. Timely referrals to mental health services and appropriate medication management can effectively address these issues.

Nouwens [19] explored the consequences of BIF on daily life, emphasizing that hurdles individuals with BIF may face in terms of education, employment, relationships, and mental health. In conclusion, BIF has far‐reaching effects on daily life, necessitating tailored and individualized assistance to tackle the challenges faced by individuals with BIF in education, employment, social relationships, and mental well‐being.

## 4. Discussion

BIF is a neurodevelopmental condition characterized by cognitive vulnerabilities and difficulties in academic achievement and adaptive functioning. Estimates suggest that the prevalence of BIF among school‐aged children ranges from 7% to 12%, influenced by various factors, including socioeconomic status and maternal stress [17], [9]. This variability underscores the critical need for tailored interventions to prevent academic underachievement and potential school dropout [8]. In line with this perspective, our findings further support the view that BIF should not be interpreted solely as an IQ‐defined condition, but as a developmental vulnerability with meaningful implications for everyday adaptive functioning [4].

The complexities of diagnosing BIF, including the presence of co‐occurring psychiatric comorbidities, necessitate an integrated approach to ensure that individuals receive adequate support. Children with BIF often experience delays in cognitive and academic skills, which require customized support and continuous monitoring [17]. Educational strategies should be individualized to align assessments with the child′s developmental level and cognitive profile, helping to avoid overly negative evaluations of their skills.

Additionally, the impact of BIF extends beyond the individual to affect families significantly. Parents may experience emotional challenges such as anxiety and frustration while navigating the complexities associated with BIF. Families often face stigma or isolation, which can exacerbate stress levels. Financially, the costs related to specialized educational resources and caregiving responsibilities can strain family budgets. Recognizing these broader implications is essential for developing comprehensive support systems that address the needs of both individuals with BIF and their families.

### 4.1. Homogeneous and Inhomogeneous Cognitive Profiles in BIF

Understanding the cognitive profiles of individuals with BIF is essential for developing effective interventions. These profiles can generally be categorized as either homogeneous or inhomogeneous, with each having distinct characteristics that align differently along the Gaussian distribution of cognitive abilities.

The concept of inhomogeneous profiles should be clearly differentiated from comorbid neurodevelopmental disorders, as the two may overlap but are not synonymous.

Homogeneous profiles are characterized by a consistent level of cognitive functioning across various domains, such as language, memory, and motor skills. Individuals with homogeneous profiles often display a flat cognitive profile, meaning their abilities are evenly distributed and fall within a narrow range. This type of profile is more closely aligned with intellectual disability, as both show a lack of variability in cognitive performance. Individuals with homogeneous profiles tend to have cognitive abilities that cluster at the lower end of the Gaussian curve, indicating generalized impairments rather than specific strengths or weaknesses. Even if their overall cognitive performance is slightly higher than that seen in intellectual disability, they may still experience significant challenges in adaptive behavior and daily functioning due to this broad, uniform level of impairment Cornoldi et al. [11].

Inhomogeneous profiles, in contrast, are marked by variability in cognitive performance across different domains. Individuals with inhomogeneous profiles may show strengths in certain areas, such as perceptual reasoning, while experiencing significant deficits in others, like working memory or language skills. This uneven cognitive functioning suggests that these individuals are positioned differently along the Gaussian curve, often showing a mix of high and low performances across various cognitive tasks. Such variability can be indicative of past neurodevelopmental challenges or specific neuropsychological deficits related to areas such as language, attention, or motor coordination [9]. Unlike those with homogeneous profiles, individuals with inhomogeneous profiles do not exhibit a uniform cognitive deficit pattern but rather a more complex profile that reflects both areas of relative strength and weakness.

Understanding these differences is critical regarding specific needs of each profile type: a broad‐based support for homogeneous profiles or the targeted interventions required for inhomogeneous ones. For individuals with inhomogeneous profiles, interventions should focus on enhancing areas where the individual struggles, such as targeted language training or motor skill improvement. In contrast, interventions for those with homogeneous profiles should be aimed at improving overall cognitive functioning and adaptive skills, like social interactions, self‐care, and problem solving.

A nuanced understanding of these cognitive profiles allows practitioners to create more targeted and effective interventions that cater to the specific needs of each individual, ultimately leading to better outcomes in their educational, social, and personal lives.

### 4.2. Regression to the Mean and Stability of Cognitive Scores

Another important consideration in the assessment of BIF is the regression to the mean phenomenon, which describes how extreme test scores tend to move closer to the average upon retesting. In the context of BIF, this means that individuals who score very high or very low on intelligence tests might score closer to the average when they are retested. This phenomenon is particularly relevant for understanding the long‐term stability of BIF diagnoses.

If a child with BIF has an exceptionally low IQ score at one point in time, their score may improve upon retesting, moving closer to the average IQ range. Recognizing this phenomenon underscores the importance of consistent monitoring and reassessment of cognitive abilities over time. A single test score may not provide a complete or accurate picture of an individual’s cognitive functioning, so ongoing assessments are necessary to track changes and adjust interventions accordingly. This approach helps ensure that individuals receive appropriate support based on their current needs rather than relying solely on initial test results.

Additionally, the stability of cognitive scores over time in individuals with BIF is a crucial factor. Some studies suggested stability in IQ scores, despite variations in fluid intelligence scores that can fluctuate in children with BIF [16] This may be due to various factors such as developmental changes, environmental influences, and the quality of interventions received. This variability highlights the need for continuous assessment to better understand the cognitive development and stability in individuals with BIF, recognizing that cognitive abilities can fluctuate and that extreme scores may not be stable.

### 4.3. Socioeconomic and Environmental Factors in BIF Development and Outcomes

Socioeconomic and environmental factors play a significant role in the development and outcomes of BIF. Individuals with BIF often come from lower socioeconomic backgrounds, limiting their access to high‐quality education, healthcare, and social services. This lack of access exacerbates cognitive and developmental delays, as these individuals may not receive early interventions that could mitigate their difficulties [17]. Children from disadvantaged backgrounds may attend underresourced schools with limited support for special educational needs, resulting in inadequate individualized educational plans (IEPs) or specialist teaching. Similarly, access to comprehensive healthcare services, including psychological and speech therapy, is often restricted in low‐income settings, further hindering their development. Social services can provide vital support through counseling, family therapy, and community programs, which help reduce stressors and improve adaptive functioning in children with BIF. Addressing these disparities through policies that ensure equitable access to education, healthcare, and social services is crucial for improving outcomes.

Environmental stressors such as high levels of stress, exposure to violence, and inadequate nutrition also impact the development of individuals with BIF. High maternal stress and adverse childhood experiences (ACEs) are linked to poorer cognitive outcomes, impairing brain development and exacerbating learning difficulties [18]. Reducing these environmental stressors is essential for fostering better cognitive and adaptive outcomes in this population.

Although intervention studies were excluded from this systematic review, it is important to outline potential approaches that may be relevant for individuals with BIF. Interventions should be tailored to their unique cognitive and developmental profiles, addressing academic, social, and behavioral challenges. Differentiated instruction, personalized learning plans, and multisensory teaching techniques may help target specific weaknesses such as phonemic awareness or arithmetic skills.

Therapeutic interventions, such as speech and language therapy, occupational therapy, and cognitive‐behavioral therapy (CBT), are valuable for improving adaptive skills, including social communication and self‐regulation. For adolescents and adults with BIF, vocational training and life skills programs are essential to support independence and enhance quality of life. Evidence from broader neurodevelopmental populations [20] indicates that multimodal educational and therapeutic programs can improve cognitive functioning and adaptive behavior; however, specific evidence in BIF remains scarce.

This highlights the importance of a comprehensive, individualized approach to intervention, while underscoring the need for future studies to directly evaluate these strategies in BIF populations.

### 4.4. Limitations

This review has several important limitations that must be acknowledged. First, despite an extensive search strategy and an updated search covering the 2024–2025 period, the final evidence base included only eight eligible empirical studies. This limited yield reflects both the scarcity of original research specifically addressing BIF in developmental age and the restrictive effect of applying rigorous eligibility criteria. As a consequence, the reliability of the conclusions is reduced and the generalizability of findings across different contexts and developmental stages remains limited.

Second, although the inclusion criteria covered the full developmental range (0–18 years), the available evidence predominantly concerned school‐aged children (6–12 years). The lack of subgroup analyses across developmental stages limits the applicability of the findings to preschool children and adolescents.

Third, definitional and diagnostic inconsistencies across studies represent a major challenge. Included samples were classified using different ICD‐10 codes and IQ‐based thresholds, leading to variability in diagnostic criteria that reduces comparability across studies and weakens conclusions regarding prevalence estimates and the delineation of cognitive profiles.

Fourth, the evidence summarized in Tables 1, 2, and 3 should be interpreted with caution due to methodological limitations in the included studies. Several investigations lacked control groups, and effect sizes and confidence intervals were inconsistently reported, limiting the robustness of group comparisons and the strength of the conclusions.

Another limitation is the absence of a quantitative synthesis. A meta‐analytic approach was not feasible due to the small number of eligible studies and the marked heterogeneity in study designs, diagnostic definitions, outcome measures, and reporting standards. Therefore, the synthesis remained qualitative, without pooled effect size estimates, formal assessment of heterogeneity, or evaluation of publication bias.

Finally, given the limited and heterogeneous evidence base, any clinical or educational implications derived from this review must be regarded as provisional and context dependent. More rigorous, standardized, and longitudinal studies are needed to strengthen the empirical foundation and support the development of clearer guidelines for assessment and management of individuals with BIF.

## 5. Conclusions

This work is aimed at highlighting the findings emerging from the available literature on BIF while stressing the numerous gaps that still characterize this field. Diagnostic criteria vary considerably across studies, but the DSM‐5 (APA 2013) recommendation to complement cognitive testing with an accurate evaluation of conceptual, social, and practical domains appears particularly useful in defining this condition and identifying possible subgroups. Such an approach requires standardized tools, clinical observation, interviews, and a careful consideration of cultural and environmental factors

BIF is a highly prevalent condition in developmental age, but literature on disposal lacks epidemiological studies that use defined inclusion criteria.

Developmental trajectories, although delayed in cognitive and academic domains, follow a course similar to that of typically developing children. This aspect can be emphasized during diagnostic counseling to reassure parents about the gradual improvements expected over time, while also underlining the importance of adequate stimulation and tailored educational and rehabilitative pathways.

Addressing the current knowledge gaps may eventually provide stronger evidence to support the development of clinical guidelines and educational practices grounded in a more nuanced understanding of the cognitive and behavioral profiles associated with BIF. However, at present, such implications should be considered speculative: the limited and heterogeneous evidence base prevents definitive conclusions about the most effective strategies or policy directions. Any recommendations for clinical or educational interventions must therefore be viewed as provisional, pending more robust empirical data.

Nevertheless, the methodological limitations of the present review—such as the small number of studies, heterogeneity in design, diagnostic criteria, and outcome measures—call for caution: the recommendations offered here should be regarded as preliminary and highly context dependent. Until more robust, longitudinal, and methodologically consistent data become available, these conclusions should be applied prudently.

## Funding

No funding was received for this manuscript.

## Conflicts of Interest

The authors declare no conflicts of interest.

## Data Availability

The data that support the findings of this study are available from the corresponding author upon reasonable request.
